# Estrogen May Enhance Toll-Like Receptor 4-Induced Inflammatory Pathways in People With HIV: Implications for Transgender Women on Hormone Therapy

**DOI:** 10.3389/fimmu.2022.879600

**Published:** 2022-06-03

**Authors:** Aaren Kettelhut, Emily Bowman, Janelle Gabriel, Brittany Hand, Namal P. M. Liyanage, Manjusha Kulkarni, Frances Avila-Soto, Jordan E. Lake, Nicholas T. Funderburg

**Affiliations:** ^1^Department of Health and Rehabilitation Sciences, The Ohio State University, Columbus, OH, United States; ^2^Department of Microbial Infection and Immunity, The Ohio State University, Columbus, OH, United States; ^3^Department of Veterinary Biosciences, The Ohio State University, Columbus, OH, United States; ^4^Department of Internal Medicine, University of Texas Health Science Center at Houston, Houston, TX, United States

**Keywords:** estrogen, inflammation, monocytes, toll-like receptor 4, human immunodeficiency virus, cardiovascular disease, transgender women

## Abstract

**Background:**

Transgender women (TW) are at increased risk for both human immunodeficiency virus (HIV) and cardiovascular disease (CVD). Antiretroviral therapy-treated HIV has been associated with a two-fold increased risk of CVD, potentially due to dysregulated Toll-like receptor (TLR)-induced immune activation. Use of estrogens in feminizing hormone therapy (FHT) may enhance inflammatory responses and the risk of cardiovascular mortality in TW. Despite this, the immunomodulatory effects of estrogen use in TW with HIV have been inadequately explored.

**Methods:**

As an *in vitro* model for FHT, cryopreserved PBMCs (cryoPBMCs) from HIV negative (HIV-), HIV+ ART-suppressed (HIV+SP), and HIV+ ART-unsuppressed (HIV+USP) cisgender men were cultured overnight in the presence of 17-β estradiol or 17-α ethinylestradiol with and without the TLR4 agonist LPS or the TLR8 agonist ssPolyU. Monocyte activation (CD69, HLA-DR, CD38) was assessed by flow cytometry. Cytokine levels (IL-6, TNF-α, IL-1β, and IL-10) were measured in cell culture supernatants by Legendplex. Levels of phosphorylated TLR signaling molecules (JNK, MAPK p38) were assessed by Phosflow. Plasma levels of immune activation biomarkers (LPS-binding protein, monocyte activation markers sCD14 and sCD163, and inflammatory molecules IL-6 and TNF-α receptor I) were measured by ELISA.

**Results:**

PBMCs from people with HIV (PWH) produced greater levels of inflammatory cytokines following exposure to LPS or ssPolyU compared to levels from cells of HIV- individuals. While estrogen exposure alone induced mild changes in immune activation, LPS-induced TLR4 activation was elevated with estrogen in cisgender men (CM) with HIV, increasing monocyte activation and inflammatory cytokine production (IL-6, TNF-α). Interestingly, testosterone inhibited LPS-induced cytokine production in CM regardless of HIV status. Plasma markers of immune activation and microbial translocation (e.g., sCD14, sCD163, LPS-binding protein) were generally higher in PWH compared to HIV- CM, and these markers were positively associated with *in vitro* responsiveness to estrogen and LPS in CM with HIV.

**Conclusions:**

Our *in vitro* data suggest that estrogen exposure may enhance innate immune activation in PWH. Further examination is needed to fully understand the complex interactions of FHT, HIV, and CVD in TW, and determine optimal FHT regimens or supplementary treatments aimed at reducing excess immune activation.

## Introduction

Transgender women (TW) are an underserved population in medicine. Treatment with gender-affirming, feminizing hormone therapy (FHT) using estrogen-based supplementation can significantly improve quality of life (1, 2). Despite the success of FHT, long-term estrogen use may contribute to increased prevalence of chronic co-morbidities including metabolic, pulmonary, cardiovascular, and immunologic complications (3–5).

Prevalence of human immunodeficiency virus (HIV) and cardiovascular disease (CVD) is higher in TW compared to the general population (6–11). TW have an approximately 3-fold greater risk of myocardial infarction (MI) compared to their cisgender counterparts (11). Additionally, MI prevalence is greater in TW receiving FHT compared to TW not on FHT (9). Studies have identified a 3-fold increased risk of cardiovascular mortality with estrogen use in TW (4). Despite these findings, mechanisms underlying FHT-associated CVD risk have been incompletely explored.

Increased CVD risk as a consequence of FHT may be particularly concerning in TW with HIV as HIV is linked to heightened CV morbidity and mortality (12). Globally, the prevalence of HIV in TW is 19% compared to < 1% in the general population; the CDC reports TW are 49 times more likely to live with HIV than cisgender women (CW) (6, 7). Current antiretroviral therapy (ART) regimens successfully manage HIV progression, however, persistent immune activation is a strong predictor of CVD in people with HIV (PWH) (13–16). Chronic immune activation may be driven by multiple factors, including the activation of Toll-like receptors (TLRs) by microbial products that translocate through damaged gut lumen or by products of low-level HIV replication (14–30). Increased monocyte and macrophage activation has been implicated in the pathogenesis of HIV-associated CVD (31–33). Exposure to bacterial (e.g., lipopolysaccharide (LPS) or flagellin) or viral products (e.g., single-stranded polyuridine (ssPolyU), imiquimod, or HIV-1) triggers cellular activation and production of pro-inflammatory mediators. Compared to those without HIV, exposure of myeloid cells from PWH to TLR ligands can result in elevated inflammatory cytokines levels including IL-6, a molecule linked to increased morbidity and mortality in PWH (34–36).

Several studies demonstrate that estrogen may enhance inflammatory responses to TLR ligands (37–41). We have measured cardiometabolic profiles and associated inflammatory biomarkers in cisgender men (CM) and TW and reported increased immune activation in the latter, regardless of HIV status. We also observed slight elevations in the CVD-associated molecules TNF-α receptor I (TNFRI), oxidized low-density lipoprotein (oxLDL), and human extracellular newly-identified receptor for advanced glycation end products binding protein (EN-RAGE) in TW on FHT compared to those off FHT (42). Further work is needed to elucidate the mechanisms that contribute to cardiometabolic risk in TW with HIV. Here, we explored the *in vitro* consequences of estrogen (17-β estradiol or 17-α ethinylestradiol) exposure to TLR-stimulated peripheral blood mononuclear cells (cryoPBMCs) from CM who were HIV negative (HIV-), HIV-positive with ART suppression (HIV+SP), or HIV-positive and ART-naïve/unsuppressed (HIV+USP) to test our hypothesis that estrogen can enhance TLR responsiveness in monocytes from PWH compared to those without HIV. While this work will be limited in its generalizability to TW, as these individuals may experience greater health disparities associated with CVD due to minority stress and marginalization when compared to CM (43), this work serves as a model to elucidate the immune modulatory effects of exogenous estrogen in those assigned male at birth with HIV.

## Materials and Methods

### Sample Collection and Cell Culture Methods

For HIV- donors, blood samples were collected by EDTA-containing Vacutainer tubes (BD Biosciences) from which PBMCs and plasma were isolated. PBMCs were freshly isolated by centrifugation over Ficoll-Hypaque and cryopreserved in freezing media made with 90% DMSO and 10% FBS at 1 ml per 1 x 10^7^ cells. To isolate plasma, EDTA tubes were centrifuged for 10 minutes at 470 g.

CryoPBMCs and plasma from ART HIV+SP (viral load < 20 copies/mL) and HIV+USP (viral load > 10,000 copies/mL) PWH were supplied by the Center for AIDS Research Network of Integrated Clinical Systems repository (CFAR CNICS). All cryopreserved cells were thawed with pre-warmed phenol red free RPMI 1640 (Gibco) that contained 10% Human Male AB OTC Serum, heat inactivated (ATCC Lot #A18049). Trypan Blue staining (1:1) was used to assess viability of thawed PBMCs. Cells were rested overnight (1 x 10^6^/ml) in 12 well culture plates before stimulating with 0.001% of ethanol (vehicle control), 1 μM of 17-β estradiol (Sigma-Aldrich), 1 μM of 17-α ethinylestradiol (Sigma-Aldrich), or 1 μM of testosterone (Sigma-Aldrich) in the absence or presence of 100 ng/ml lipopolysaccharide (Sigma-Aldrich) or 1 μg/ml ssPolyU (*In vivo*gen) overnight in 37°C incubator with 5% CO_2_. All hormones were obtained in powder form and solubilized in 100% ethanol before filtering. Concentrations of hormones were selected based on initial dose response assays. For phosphoflow experiments, cells were exposed to 0.001% of ethanol or 1 μM of 17-β estradiol in the presence or absence of 100 ng/ml lipopolysaccharide for 20 minutes in 37°C incubator before cell collection.

### Flow Cytometry

Cells were collected and supernatants stored in -80°C for cytokine analysis. Cells were washed in 1X Dulbecco Phosphate Buffered Saline (PBS; Gibco) followed by flow wash buffer. Cells were stained for 15 minutes at room temperature in the dark, washed, and fixed in 1% paraformaldehyde (Thermoscientific). Monocytes were identified by size, granularity, and surface expression of CD14 (anti-CD14 Pacific Blue or Pacific Blue isotype; BD Biosciences Cat# 558121, RRID : AB_397041 or BD Biosciences Cat# 558118, RRID : AB_397039, respectively). Monocyte activation markers were measured using anti-CD38 (phycoerythrin [PE]; BD Biosciences Cat# 555460, RRID : AB_395853), anti-CD69 (PE-Cyanine7 [PeCy7]; BD Biosciences Cat# 335792, RRID : AB_1937286), anti-HLADR (APC-Cyanine7 [APCCy7]; BD Biosciences Cat# 335814, RRID : AB_399991) or their respective isotypes (BD Biosciences Cat# 555749, RRID : AB_396091; BD Biosciences Cat# 348798, RRID : AB_400386) by Miltenyi MACSQuant Analyzer 10 flow cytometer (Miltenyi Bioscience MACSQuant Analyzer 10, RRID : SCR_020268) and MACSQuant analysis software (MACSQuantify, RRID : SCR_020943).

### Phosphoflow Cytometry

Cells were collected and washed in PBS and flow wash buffer, similar to above protocol, then stained for surface expression of CD14 (anti-CD14 V500 or isotype; BD Biosciences Cat# 561392, RRID : AB_10611862 or BD Biosciences Cat# 561221, RRID : AB_10566127 respectively) for 20 minutes in the dark at 37°C. Monocyte intracellular expression of phosphorylated proteins were measured using an intracellular staining protocol. Cells were lysed at 37°C for 10 minutes with lyse/fix buffer (BD Phosflow), washed with PBS, and permeabilized at 4°C for 30 minutes with Perm Buffer III (BD Phosflow). Cells were washed 3 times in stain buffer (BD Phosflow) before staining in the dark at room temperature for 1 hour with anti-JNK (pT183/pY185) (PE; BD Biosciences Cat# 562480, RRID : AB_11153134) and anti-p38 MAPK (pT180/pY182) (peridinium-chlorophyl-protein Complex-Cyanine 5.5 [PerCP-Cy5.5]; BD Biosciences Cat# 560406, RRID : AB_164529). Cells were analyzed by the MACSQuant Analyzer 10 and analysis software. Monocytes were again identified by size, granularity, and surface expression of CD14.

### RNA Isolation, cDNA Conversion, and Quantitative Polymerase Chain Reaction

Cells were stimulated as described above with 0.001% of ethanol, 1 μM of 17-β estradiol, or 1 μM of 17-α ethinylestradiol in the absence or presence of 100 ng/ml lipopolysaccharide or 1 μg/ml ssPolyU for 24 hours in the 37°C incubator. Cells were collected, washed in PBS, and placed in 1% 2-Mercaptoethanol (Sigma Aldrich) supplemented lysing buffer (Qiagen) and stored at -80°C overnight for optimal cell lysing. Cell supernatants were collected for cytokine analysis and stored at -80°C. RNA was extracted using the RNeasy Mini Kit (Qiagen) and RNase-Free DNase set (Qiagen). Extracted RNA was converted to cDNA using the iScript cDNA Synthesis Kit (Bio-rad) and S1000 thermal cycler (Bio-rad). Resultant cDNA along with IQ Sybr Green Supermix (Bio-rad) and relevant forward and reverse primers were used for qPCR analysis. This analysis assessed expression of cytokines IL6 (F: 5’CCAGGAGCCCAGCTATGAAC 3’, R: 5’CCCAGGGAGAAGGCAACTG 3’) and TNF-α (F: 5’GAGGCCAAGCCCTGGTATG 3’, R: 5’CGGGCCGATTGATCTCAGC 3’) utilizing MicroAmp Optical 6-well reaction plates and Real-Time PCR System Quant Studio 3 (Applied Biosystems; QuantStudio 3 Real Time PCR System, RRID : SCR_018712). Results were analyzed using QuantStudio design and analysis software.

### MAPK p38 Inhibition

Cryopreserved PBMCs were rested overnight (1 x 10^6^/ml) in 12 well culture plates before stimulating with 0.001% of ethanol (vehicle control) or 1 μM of 17-β estradiol (Sigma-Aldrich) in the absence or presence of 100 ng/ml lipopolysaccharide (Sigma-Aldrich) with or without 10 µM of p38 inhibitor SB203580 (Sigma-Aldrich) overnight in 37°C incubator with 5% CO_2_. Supernatants were collected for cytokine analysis and stored at -80°C.

### Measurement of Cytokine Expression

Levels of inflammatory cytokines were measured in supernatants using the LEGENDplex HU Anti-virus response panel (13-plex) w/VbP (Biolegend). Supernatants collected from cells stimulated with ssPolyU or no stimulation were diluted 1:5 in provided assay buffer while those stimulated with LPS were diluted 1:100. LEGENDplex was run on MACSQuant 10 flow cytometer and analyzed by the LEGENDplex Data Analysis Software.

### Plasma Biomarker Assessment

Plasma biomarker levels were measured by enzyme-linked immunosorbent assay (ELISA; R&D unless otherwise stated) using a SpectraMax 190 plate reader (SpectraMax 190 microplate reader, RRID : SCR_018932). These biomarkers include soluble CD14 (sCD14), soluble CD163 (sCD163), tumor necrosis factor receptor 1 (TNFR-1), high sensitivity interleukin 6 (HS IL6), LPS-binding protein (LBP, Hycult Biotech), and β D-glucan (BDG, MyBioSource). Data was collected in SoftMax Pro 7.0.2 (SoftMax Pro Data Acquisition and Analysis Software, RRID : SCR_014240).

### Statistical Analysis

Statistical analysis for flow, gene expression, and cytokine analyses were performed in GraphPad Prism 8 (GraphPad Prism, RRID : SCR_002798) utilizing nonparametric paired and unpaired *t* tests along with nonparametric 1- way ANOVA analysis to compare data between our 3 donor groups with our different stimulations. Statistical analysis for ELISA and demographic data were also performed in GraphPad Prism 8 utilizing nonparametric 1- way ANOVA analysis to compare clinical and plasma biomarker data between donor groups. Spearman correlations were utilized to assess relationships between measured plasma biomarkers, plasma biomarkers and clinical data, and plasma biomarkers and changes in cytokine production levels. Correlation heatmaps were created with R software to assess relationships between clinical information, baseline immunoinflammatory profiles, and cytokine responses.

## Results

### Plasma Biomarkers of Immune Activation and Microbial Translocation are Elevated in PWH

Several studies, including our own, have reported increased levels of pro-inflammatory molecules in PWH and in TW on and off FHT compared to levels in CM, regardless of HIV status. Here, we explored the *in vitro* effects of estrogen on TLR-induced monocyte activation profiles in PWH. As a model for *in vivo* processes in TW initiating FHT, we utilized cryoPBMCs from 18 CM not known to have HIV and 29 CM with HIV, including individuals with HIV+SP (n=14) and HIV+USP (n=15) viral replication. Median viral loads (20 copies/mL and 98,138 copies/mL) and CD4^+^ T-cell counts (708 and 334 cells/μL) were significantly different in HIV+SP and HIV+USP participants (p<0.001). While median age was not significantly different among groups (HIV- 45.0; HIV+SP 50.5; HIV+USP 49.0, p>0.05), racial identification was different between HIV- individuals and PWH, with more White and Asian participants among the former group (p<0.01) (**Table 1**). Participants were approved for enrollment under the IRB protocol ID 2019H0113.

**Table 1:** Baseline clinical characteristics and biomarkers of immune activation and inflammation in plasma of HIV- and HIV+ antiretroviral therapy-suppressed and -unsuppressed donors.

Clinical Data & Biomarkers	CM, HIV- (n=11)	CM, HIV+ ART-suppressed (n=14)	CM, HIV+ ART-unsuppressed (n=15)	P value
**Age (years)**	45.0 (25, 68)* ^(17)^	50.5 (23, 60)	49.0 (23, 55)	p=0.8932
**Race (%)^±^**				**p=0.003**
** White**	61.1%* ^(18)^	35.7%	40.0%	–
** Black**	11.1%* ^(18)^	64.3%	60.0%	–
** Asian**	27.8%* ^(18)^	0.0%	0.0%	–
**Viral Load (copy/mL)**	N/A	20	98138 (13464, 443509)	**p<0.0001**
**CD4 T cell count (cells/μL)**	N/A	708 (315, 1253)	334 (12, 866)	**p=0.0002**
**BDG (pg/mL)**	310.35 (236.33, 456.76)* ^(8)^	273.94 (175.08, 904.11)* ^(12)^	287.04 (191.17, 426.24)	p=0.5438
**LBP (ng/mL)**	12332.9 (5129.84, 15806.41)	15881.42 (4306.69, 28272.54)	16233.13 (4.4, 26356.45)	p=0.2816
**IL-6 (pg/mL)**	1.53 (0.43, 3.16)	1.96 (0.366, 22.18)	2.71 (0.818, 9.321)	p=0.2069
**sCD14 (pg/mL)**	1616.31 (1000.01, 1756.68)	1662.94 (1077.22, 2544.94)	1948.18 (974.50, 2573.15)	**p=0.0186**
**sCD163 (ng/mL)**	502.89 (265.19, 800.31)	620.35 (337.24, 1451.93)	1233.86 (473.86, 2668.30)	**p=0.0004**
**TNFRI (pg/mL)**	1145.50 (794.34, 1440.69)	1072.40 (686.92, 1966.95)	1298.50 (764.51, 2064.37)	p=0.1308

Median values (and range) reported. Biomarker data was obtained from plasma samples of participants, if available, utilizing enyzme linked immunosorbent assay. CM, cisgender men; ART, antiretroviral therapy; BDG, β-d-Glucan; LBP, lipopolysaccharide binding protein; IL-6, interleukin-6; sCD14, soluble CD14; sCD163, soluble CD163; TNFRI, TNF-α Receptor I; N/A, not applicable; *# represents if the n is different than listed where *(#), with # representing any number, indicates n=# as a result of plasma or clinical data availability; significance by one-way ANOVA or ^±^ chi-square test. Significant data bolded (p<0.05).

Plasma biomarkers were measured to assess *in vivo* immune activation in participants with available samples (HIV- n=11, HIV+SP n=14, HIV HIV+USP n=15, **Table 1**), including potential drivers of inflammation in PWH (BDG and LBP), inflammatory molecules (IL-6, TNFRI), and monocyte activation markers (sCD14, sCD163). Of these, only CD14 and CD163 were significantly different between groups (p<0.05, p<0.001). *Post hoc* analysis revealed significant differences between HIV- and HIV+USP for both markers (CD14 p<0.05, CD163 p<0.001) and between HIV+SP and HIV+USP for CD163 (p<0.05). TNFRI was elevated in USP donors, while IL-6 and LBP increased in both groups with HIV compared to HN participants (p>0.05).

### Antiretroviral Therapy Suppressed PWH Demonstrate Significant Positive Associations Between Markers of Immune Activation and LPS-Binding Protein

**Figure 1:**
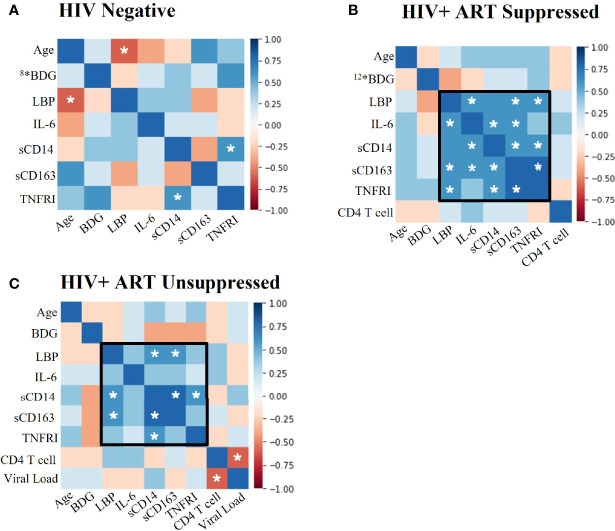
Plasma biomarkers show significant positive associations between LBP and immunoinflammatory markers in PWH. Spearman Rank Correlation between clinical and plasma biomarker data in **(A)** HIV negative (n=11), **(B)** HIV+ ART-suppressed (n=14), and **(C)** HIV+ ART-unsuppressed (n=15) donor groups. PWH, people with HIV; ART, antiretroviral therapy; BDG, β-d-Glucan; LBP, lipopolysaccharide binding protein; IL-6, interleukin-6; sCD14, soluble CD14; sCD163, soluble CD163; TNFRI, TNF-α Receptor I; *#, where # represents any number, indicates a different n than listed where #* is equivalent to n=# due to issues in plasma availability; white stars represent significance of p<0.05.

We assessed correlations among biomarkers and contributing mediators of immune activation in PWH to better understand activation profiles in participant groups. We found significant positive associations among LBP, IL-6, TNFRI, CD14, and CD163 in HIV+SP donors (p<0.05, **Figure 1B**). These associations, while not all statistically significant, remained in the HIV+USP group. Viral loads and CD4^+^ T cell counts were negatively associated in HIV+USP donors (p<0.05); however, viral loads were not significantly associated with markers of inflammation (**Figure 1C**). Fewer associations were seen in HIV- donors, with only one association reaching significance (p<0.05, **Figure 1A**). Age and LBP were significantly and negatively associated in HIV- donors, otherwise age was not significantly associated with immune activation in any participant group.

### PWH Enhance Responses to Toll-Like Receptor Agonism

**Figure 2:**
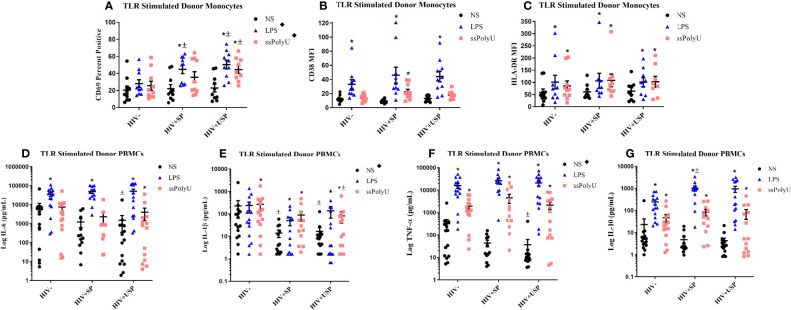
Toll-like receptor stimulation increases monocyte activation markers and cytokine production with greater increases in PWH. HIV+ ART-suppressed (HIV+SP), HIV+ ART-unsuppressed (HIV+USP), and HIV negative (HIV-) cryopreserved peripheral blood mononuclear cells (PBMCs) from CM were thawed and rested overnight in phenol red free RPMI supplemented with 10% Human AB Serum. Cells were treated overnight with no stimulation (NS), 100 ng/mL of TLR4 agonist LPS, or 1 μg/mL of TLR8 agonist ssPolyU. **(A–C)** Cells were collected and stained for CD14+ monocytes and analyzed for levels of relevant activation markers (CD69, CD38, HLA-DR) expressed as percent positive cells or mean fluorescent intensity (MFI) (n=10 all groups). **(D–G)** Supernatants were collected for protein analysis of inflammatory cytokines (IL-1β, IL-6, TNF-α, IL-10) by multiplex bead assay (HIV- n=15, HIV+SP n=12, HIV+USP n=15). Data is represented as mean and standard error of measure in **(A–C)** raw or **(D–G)** log format and was analyzed by *Wilcoxon signed rank test, ^±^ Mann Whitney U, or ^♦^one-way ANOVA (*^,±,♦^p<0.05). CM, cisgender men; ART, antiretroviral therapy; LPS, lipopolysaccharide; ssPolyU, single stranded polyuridine; TLR, Toll-like receptor; NS, no stimulation; PWH, people with HIV; IL, interleukin; TNF, tumor necrosis factor.

Alterations in TLR expression and function have been reported in PWH (36, 44, 45). We examined the effects of TLR ligands on monocyte activation markers (CD69, HLA-DR, CD38, n=10 all groups) and cytokine production (IL-6, TNF-α, IL-1β, IL-10, HIV- n=15, HIV+SP n=12, HIV HIV+USP n=15) from cryoPBMCs. The proportion of monocytes that expressed CD69 and the mean fluorescence intensities (MFIs) of HLA-DR and CD38 were increased in all groups following exposure to LPS or ssPolyU. TLR-induced expression of activation markers tended to be higher in PWH compared to expression on cells from people without HIV (**Figures 2A–C**). Exposure of cells to LPS or ssPolyU also increased production of inflammatory cytokines from all participant groups. Despite higher baseline cytokine production in HIV- participants, LPS exposure resulted in greater production of cytokines from the cells of PWH compared to cells from HIV- individuals (**Figures 2D–G**).

### 17-β Estradiol Enhances LPS-Induced Activation Profiles in PWH

Estrogen may enhance TLR expression and function (37), therefore, we assessed alteration in cellular responses to LPS stimulation with estrogen treatment in our participants. Exposure of cells to either 17-β estradiol (17B) or 17-α ethinylestradiol (17A) alone inconsistently reduced expression of activation markers on monocytes from all participant groups (**Supplemental Figures 1A–C**). Exposure of cells from PWH to 17B, however, increased cytokine production, with significantly higher IL-6 production from HIV+SP versus HIV- cells (p<0.05, **Supplemental Figures 1D–G**).

**Figure 3:**
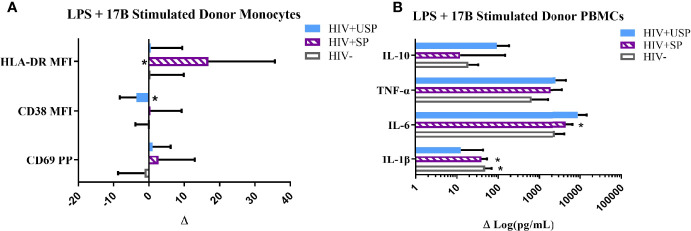
Estrogen differentially modulates monocyte activation and cytokine production with LPS stimulation. HIV+ ART-suppressed (HIV+SP), HIV+ ART-unsuppressed (HIV+USP), and HIV negative (HIV-) cryopreserved peripheral blood mononuclear cells (PBMCs) from CM were thawed and rested overnight in phenol red free RPMI supplemented with 10% Human AB Serum. Cells were treated overnight with 0.001% ethanol (vehicle) or 1 μM of either 17β estradiol (17B) (17A) +/- 100 ng/mL of LPS. **(A)** Cells were collected and stained for CD14+ monocytes and analyzed for levels of relevant activation markers (CD69, CD38, HLA-DR) expressed as percent positive cells or mean fluorescent intensity (MFI) (n=10). **(B)** Supernatants were collected for protein analysis of inflammatory cytokines (IL-1β, IL-6, TNF-α, IL-10) by multiplex bead assay (HIV- n=15, HIV+SP n=12, HIV+USP n=15). Data is normalized to LPS + vehicle and is represented as delta change (Δ) of marker or cytokine expression with mean and standard error of measure shown. Data in **(B)** represented in log format. Data was analyzed by *Wilcoxon signed rank test which assessed for significant differences in activation marker expression or cytokine production between LPS + vehicle and LPS + 17B stimulation for each donor group (*p<0.05). Mann Whitney U and one-way ANOVA analyses were also run, but no significant differences were found. CM, cisgender men; ART, antiretroviral therapy; LPS, lipopolysaccharide; IL, interleukin; TNF, tumor necrosis factor.

Based on these results, and as 17B is the preferred estrogen for FHT (46), we focused on the effects of 17B in follow-up experiments. In combination with LPS, estrogen exposure typically enhanced monocyte activation marker expression in HIV+SP participants. HLA-DR expression levels were increased significantly in HIV+SP participants with the combination of estrogen and LPS compared to expression induced by LPS alone (p<0.05; **Figure 3A**). Cells from all groups increased cytokine production with combined treatment compared to LPS alone; IL-6 production was significantly increased with combined stimulation in HIV+SP PWH (p<0.05, **Figure 3B**). Greater production of IL-6 and TNF-α was seen in PWH compared to HIV- participants. Changes in mRNA expression of IL-6 and TNF-α reflected results seen at the protein level (**Supplemental Figures 2–4**). For comparison, we tested cryoPBMC responses to testosterone in the absence or presence of LPS. Combined treatment with LPS showed increases in activation marker expression on monocytes from all groups (**Supplemental Figures 5A, B**). Cytokine production tended to increase with testosterone alone - albeit to a lesser degree than estrogen alone for IL-6 and TNF-α – but testosterone decreased cytokine production when combined with LPS in all groups compared to levels from cells exposed to LPS alone (**Supplemental Figures 5C, D**).

### *In Vivo* Activation Profiles May Influence *Ex Vivo* Responsiveness to Estrogen and TLR Stimulation in PWH

**Figure 4:**
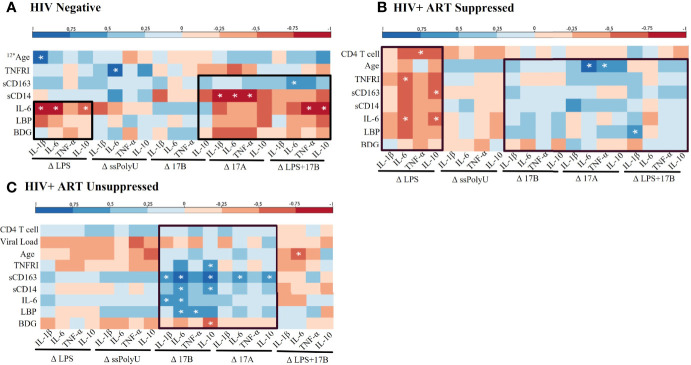
Baseline *in vivo* inflammatory profiles may play a role in response to *in vitro* stimulations. Spearman Rank Correlation between clinical or plasma biomarker data and delta change (Δ) in cytokine production, determined by ELISA or bead assay respectively, by TLR and/or estrogen stimulation in **(A)** HIV negative (n=8), **(B)** HIV+ ART-suppressed (n=12), and **(C)** HIV+ ART-unsuppressed (n=15) donor groups. ART, antiretroviral therapy; LPS, lipopolysaccharide; ssPolyU, single stranded polyuridine; 17B, 17β estradiol; 17A, 17α ethinylestradiol; BDG, β-d-Glucan; LBP, lipopolysaccharide binding protein; IL-6, interleukin-6; sCD14, soluble CD14; sCD163, soluble CD163; TNFRI, TNF-α Receptor I; IL, interleukin; TNF, tumor necrosis factor; ELISA, enyzme linked immunosorbent assay. #* represents different n than listed where #* is equivalent to n=# due to issues in plasma availability *p<0.05.

We next examined relationships between *in vivo* profiles of inflammation and immune activation in our participant groups and the *ex vivo* responsiveness of PBMCs to TLR and estrogen stimulations. LPS-stimulated HIV- PBMCs showed negative associations between plasma IL-6 and LBP levels and *ex vivo* cytokine production (**Figure 4A**). Negative associations were also seen among several plasma biomarkers and LPS-induced cytokine production in our HIV+SP group (**Figure 4B**). In PWH, positive associations were observed between estrogen-induced cytokine production and participant activation profiles (**Figures 4B, C**). In those without HIV, however, estrogen-induced cytokine production had mixed associations with activation profiles. The relationships seen between estrogen-induced cytokine production and immune activation profiles remained consistent with the addition of LPS in both HIV- and HIV+SP participant groups. Combined LPS- and estrogen-induced cytokine production in HIV+USP participants no longer showed positive or significant associations with immune activation profiles.

### Inhibition of MAPK p38 Abates 17β Enhanced LPS-Induced Inflammatory Responses

**Figure 5:**
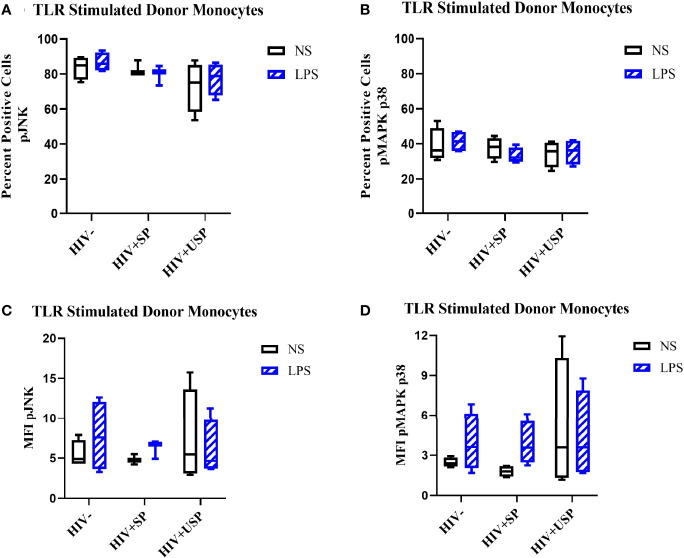
LPS increases phosphorylation levels of JNK and p38 in HIV- and HIV+USP, but not HIV+SP, participants. HIV+ ART-suppressed (HIV+SP), HIV+ ART-unsuppressed (HIV+USP), and HIV negative (HIV-) cryopreserved peripheral blood mononuclear cells (PBMCs) from CM were thawed and rested overnight in phenol red free RPMI supplemented with 10% Human AB Serum. Cells were treated for 20 minutes with no stimulation or 100 ng/mL of LPS. Cells were collected and stained for CD14+ monocytes and analyzed for intracellular levels of **(A, C)** phosphorylated c-Jun NH2-terminal kinase (n=4 all except HIV+SP n=3) or **(B, D)** mitogen activated protein kinase mammalian p38 (n=4 all) (pJNK or pMAPK p38 respectively) expressed as **(A, B)** percent positive cells or **(C, D)** MFI. Data is represented as mean and standard error of measure and was analyzed by Wilcoxon signed rank test, Mann Whitney U, or one-way ANOVA although no significant differences were found. CM, cisgender men; ART, antiretroviral therapy; LPS, lipopolysaccharide; TLR, Toll-like receptor; NS, no stimulation; MFI, mean fluorescent intensity.

**Figure 6:**
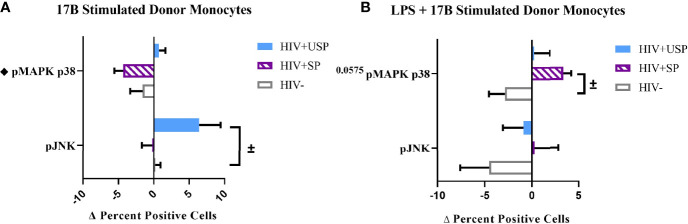
Phosphorylation of MAPK p38 in HIV+SP participants is upregulated with 17B treatment in the presence of LPS, but not alone. HIV+ ART-suppressed (HIV+SP), HIV+ ART-unsuppressed (HIV+USP), and HIV negative (HIV-) cryopreserved peripheral blood mononuclear cells (PBMCs) from CM were thawed and rested overnight in phenol red free RPMI supplemented with 10% Human AB Serum. Cells were treated for 20 minutes with 0.001% ethanol (vehicle) or 1 μM of either 17β estradiol (17B) **(A)** alone (n=4 all except HIV+SP n=3) or **(B)** with 100 ng/mL of LPS (n=4 all). Cells were collected and stained for CD14+ monocytes and analyzed for intracellular levels of phosphorylated c-Jun NH2-terminal kinase or mitogen-activated protein kinase mammalian p38 (pJNK or pMAPK p38) expressed as delta change (Δ) in percent positive cells. Data is represented as mean and standard error of measure and normalized to vehicle control **(A)** alone or **(B)** with 100 ng/mL of LPS. Data was analyzed by ^±^Mann Whitney U or ^♦^one-way ANOVA (^±,♦^p<0.05). CM, cisgender men; ART, antiretroviral therapy; LPS, lipopolysaccharide; TLR, Toll-like receptor.

**Figure 7:**
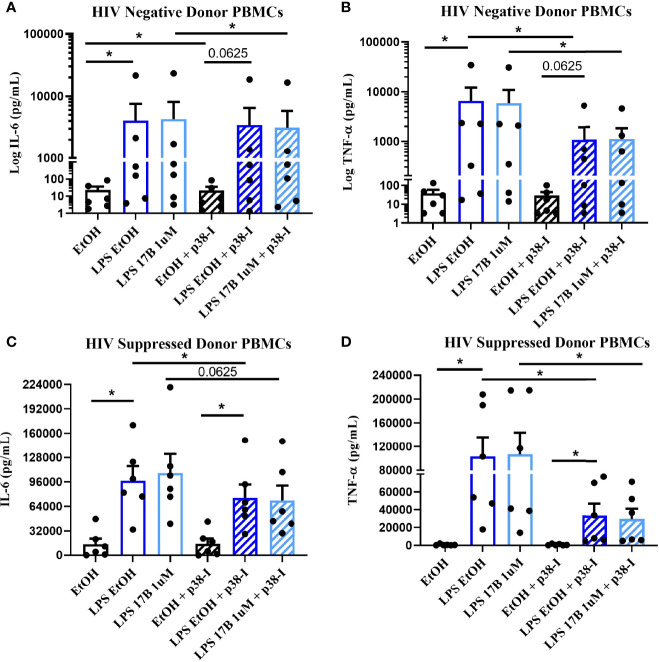
Inhibition of MAPK p38 in the presence of LPS and 17B may downregulate IL-6 and TNF-α production in ART-suppressed participants. 6 HIV+ ART-suppressed and HIV negative cryopreserved peripheral blood mononuclear cells (PBMCs) from CM were thawed and rested overnight in phenol red free RPMI supplemented with 10% Human AB Serum. Cells were treated overnight with 0.001% ethanol (vehicle) or 1 μM of 17β estradiol (17B) +/- 100 ng/mL of LPS in the presence or absence of MAPK p38 inhibitor SB203580 10 µM (p38-I). Supernatants were collected for protein analysis of inflammatory cytokines **(A, C)** IL-6 or **(B, D)** TNF-α by multiplex bead assay. Data presented in **(A, B)** log or **(C, D)** raw format and was analyzed by *Wilcoxon signed rank test. CM, cisgender men; ART, antiretroviral therapy; LPS, lipopolysaccharide; IL, interleukin; EtOH, ethanol; TNF, tumor necrosis factor.

Estrogen may modulate activation of intracellular signaling including the phosphorylation of MAPK p38 and JNK, molecules that influence inflammatory responses to TLR stimulation (47–49). We analyzed phosphorylation of these signaling molecules in the monocytes of our participant groups in the presence or absence of TLR4 stimulation. Although not statistically significant, HIV- individuals on average showed upregulation of both the proportion of monocytes that had phosphorylated JNK and MAPK p38, as well as the MFI of these phosphorylated molecules, while variable responses were seen in the cells of HIV+SP and HIV+USP participants exposed to LPS (**Figure 5**). Treatment with 17B alone or in combination with LPS showed significant alterations in the percent positive (PP) monocytes for phosphorylated JNK (pJNK) in HIV- and HIV+USP individuals, while minor changes were induced in HIV+SP participants. Phosphorylated MAPK p38 (pMAPK p38) showed decreased or little change in the PP monocytes with 17B treatment alone, however, the addition of 17B to LPS stimulation significantly increased pMAPK p38 in HIV+SP participants when compared to HIV- individuals (p<0.05, **Figure 6**). As previously demonstrated, 17B stimulation enhances LPS-induced production of IL-6 and TNF-α to a greater degree in PWH (**Figure 3**). To further understand the mechanism by which estrogen may enhance inflammatory responses and the importance of MAPK p38 signaling in PWH, we inhibited MAPK p38 activation with SB203580. Inhibition of MAPK p38 decreased LPS-induced cytokine production in both HIV- and HIV+ SP groups (**Figure 7**). Inhibition appeared to also reduce estrogen’s synergistic effects on IL-6 and TNF-α production with LPS stimulation, compared to production with LPS alone, in HIV+SP individuals (**Figures 7C, D**). The role of p38 activation in estrogen-induced cytokine production requires further study.

## Discussion

Transgender women are a clinically unique population who are especially vulnerable to both HIV and CVD. As HIV-induced chronic immune activation and inflammation can contribute to CVD risk, and estrogen exposure may exacerbate inflammatory responses, research is needed to elucidate the effects of FHT in TW with HIV.

ART reduces AIDS morbidity and mortality in PWH. Despite its ability to suppress viral replication to undetectable levels and allow restoration of peripheral CD4^+^ T cell counts in most individuals, gut permeability is often persistently altered in PWH due to incomplete restoration of CD4^+^ T cells in the gut mucosa. LPS, a bacterial product which can translocate through the gut lining, may drive HIV-induced inflammatory states through activation of myeloid cells by TLR4 ligation (50). As expected, levels of LPS-binding protein, monocyte activation markers, and inflammatory molecules tended to be increased in PWH compared to levels in people without HIV (51–53). In this study, significant positive correlations among markers of monocyte activation, inflammation, and our marker of microbial translocation were identified within PWH, relationships that were not seen among the HIV- group, signaling that microbial translocation may play an influential role in altering immune activation in this cohort.

The expression and function of TLRs are thought to be altered in PWH due to chronic exposure to bacterial and viral products from microbial translocation, HIV replication, or co-infections. In this cohort, exposure of PBMCs to either TLR4 or TLR8 agonists, LPS and ssPolyU, increased inflammatory cytokine production and monocyte activation marker expression regardless of HIV serostatus. TLR-induced activation was notably higher in PWH despite reduced baseline levels. Altered immune responses to microbial products may have arisen in these individuals after long-term exposure to chronic inflammatory stimuli prompting epigenetic or metabolic reprogramming of innate immune cell responses, a phenomenon known as trained immunity. In PWH, trained immunity may optimize inflammatory responses to successive pathogens; in the setting of chronic TLR activation, however, a persistently elevated inflammatory response may promote pathways associated with cardiometabolic risk (54, 55). Additional studies are needed to further assess the role of trained immunity in chronic HIV infection.

Several *in vivo* markers of monocyte activation and inflammation were inversely related to levels of cytokine production following *in vitro* LPS exposure among ART-suppressed PWH in our cohort. LPS desensitization, or reduced responsiveness to repeated exposure, in myeloid cells is an adaptive response to limit inflammation during chronic LPS exposure (56). Similar trends existed in HIV- individuals with elevated immune activation and LBP compared to those with reduced profiles of immune activation. While similar relationships were not consistently seen in HIV+USP individuals, there may be a unique interplay between high levels of HIV replication and products of microbial translocation that should be considered. Viral and bacterial products may trigger independent immune pathways and may differentially alter functional responses to TLR stimuli.

Chronic exposure to inflammatory stimuli *in vivo* may play a role in producing altered functional responses to TLR ligation. Studies of endogenous estrogen in human and animal models suggest estrogen modulates inflammation and TLR responsiveness (37). We hypothesized that estrogen exposure to HIV- cells would result in downregulation of immune activation, as estrogen may play an atheroprotective role, but these effects could be reversed in PWH due to functional reprogramming of cells after exposure to chronic inflammatory environments *in vivo*. While estrogen had mild effects on cellular activation in all donor groups, the directionality of the response to estrogen differed by HIV serostatus, with increases in inflammatory cytokines in PWH. Interestingly, estrogen’s atheroprotective role in HIV- individuals may be reflected by the reduced cytokine production in those with greater *in vivo* activation profiles. In contrast, PWH tended to increase inflammatory cytokines in response to estrogen in individuals with greater *in vivo* immune activation, indicating a potential shift in the cellular response to estrogen with concurrent HIV infection.

Studies of HIV in CW have revealed faster progression to AIDS compared to men with HIV and similar viral loads, as well as altered levels of plasma markers of microbial and immune activation after treatment among CW versus CM with HIV (57, 58). These differences may be explained by estrogen’s ability to modulate TLR-mediated pro-inflammatory pathways, which contribute to chronic immune activation associated with CV morbidity and mortality (57–61). The addition of 17-β estradiol tended to enhance LPS-induced expression of the activation marker HLA-DR on monocytes, and production of cytokines IL-6 and TNF-α from the PBMCs of PWH regardless of ART status; enhancement of these molecules by the addition of estrogen was reduced in those without HIV. Among HIV- individuals, increased immune activation was inversely associated with LPS-induced cytokine production following estrogen exposure, while cytokine production was directly related to *in vivo* immune activation in PWH. In the latter group, this effect differs from the indirect relationship seen with LPS alone indicating the presence of estrogen may alter functional desensitization to LPS in suppressed individuals with greater activation profiles. Previous cross-sectional biomarker data identified increased levels of inflammatory molecules associated with CVD risk, such as EN-RAGE, oxLDL, and TNFRI, in TW both on and off FHT compared to CM (42). In the presence of estrogen, elevated immune activation profiles in TW with HIV may indicate risk for enhanced inflammatory responses associated with CVD.

Estrogen can modulate the phosphorylation, and activation, of important intracellular signaling molecules in the TLR pathway including JNK and MAPK p38 (48, 49). Both of these kinases can induce upregulation of transcription factors associated with immune activation. While phosphorylation of JNK seemed to play a minor role in either HIV- or suppressed donors, phosphorylation of MAPK p38 in the presence of LPS stimulation was induced by 17-β estradiol in only ART-suppressed individuals. This may suggest that estrogen can upregulate activation of important TLR4 signaling molecules, and subsequent inflammation, during suppressive ART, but not among HIV- individuals. Inhibition of MAPK p38 normalized LPS and estrogen-induced cytokine production to levels seen with LPS alone in HIV+ SP, suggesting that MAPK p38 may play a partial role in additive effects of estrogen and LPS on cytokine production. While MAPK p38 phosphorylation in ART-suppressed individuals may be one mechanism by which estrogen alters HIV-induced inflammatory pathways, estrogen’s effects are likely complex and multifactorial, as many factors are hypothesized to influence the effects of endogenous estrogen in humans. Multiple cellular receptors have been identified for estrogen, but their functions are incompletely understood. Further research is needed to unravel the intersections among estrogen, TLRs, and drivers of inflammation in PWH.

Limitations exist in this study, including the small number of participants. As an exploratory study, these numbers were satisfactory in revealing trends that require further elucidation. Another limitation of this study was the use of cryopreserved PBMCs, as baseline activation of cells may be elevated, although cell viability was not a concern. To mitigate this issue, cells were rested overnight with supplemented media and human serum. A dose-response study was utilized to select a concentration for estrogen. While the dose chosen was greater than physiological estrogen levels, higher levels of estrogen are associated with more anti-inflammatory responses (37) furthering our hypothesis that estrogen use in biological males may have altered effects on inflammation. Due to limitations in human subject enrollment from COVID-19 restrictions, we utilized cells from CM to best replicate the biological environment present in TW pre-FHT. While a convenient solution, studies would better represent this population with cells from TW, as these individuals have increased risk for co-morbidities such as obesity, diabetes, lipid dysregulation, and CVD regardless of HIV. Further study is also needed to understand the full effects of FHT, as FHT can utilize both estrogens and anti-androgens that block the effects of testosterone. We report here that testosterone may play a role in lowering LPS-induced inflammation in PWH. Despite these factors, our findings demonstrate the need for clinical studies aimed at understanding the mechanistic role of estrogen to reduce CV mortality in TW with HIV.

Overall, this work suggests complex interactions may exist among HIV, CVD, and FHT in TW. As PWH are living longer, both increased frequency and severity of multiple end-organ diseases, such as CVDs, have been uncovered. While estrogen is a known modulator of TLR expression and function, the intensified responses to TLR activation with estrogen treatment in PWH cannot be fully explained by this phenomenon when compared to responses in HIV- individuals. This work demonstrates a unique synergistic effect of microbial-and-estrogen-induced immune activation in PWH independent of the increased activation seen with TLR ligation or estrogen treatment alone. Our findings may reflect alterations in cellular response due to chronic inflammatory stimuli that result in enhanced inflammatory pathways associated with CV morbidity.

To appropriately address clinical disparities in TW, current research assessing treatments aimed to reduce pivotal drivers of inflammation and downstream immune signaling underlying HIV-induced co-morbidities should be considered. As previously reviewed, studies range from altering the microbiota in the gut to reduce translocation of microbial products, to blocking the actions of important inflammatory molecules associated with HIV morbidity and mortality such as IL-6 (62). A multi-pronged approach with ART may be a beneficial route to provide equitable care. By understanding the mechanism behind estrogen-induced modulation of TLR pathways, we may identify important clinical indications for additional therapies in TW on FHT or identify alternative estrogen-like drugs designed to reduce deleterious immune effects in TW with HIV.

## Contributions

Estrogen has been recognized as an important modulator of immune activation including alterations in inflammation and Toll-like receptor function in innate immune cells. Mechanisms by which estrogen exerts these effects are incompletely understood and may depend on estrogen concentration, cell type, and cellular environments. While the use of estrogen in feminizing hormone therapy can improve quality of life in transgender women, research is needed to understand the side effects of long-term high dose usage. This is particularly concerning in the setting of HIV-associated chronic inflammation as Toll-like receptor 4 activation is linked to elevated systemic lipopolysaccharide and inflammation. Based on current literature, we propose that estrogen may upregulate immune activation of myeloid cells in people with HIV as a result of Toll-like receptor functional changes. The elevated immune activation seen here has been associated with increased risk for co-morbidities such as cardiovascular disease and higher mortality rates in people with HIV. As estrogen use in transgender women has been linked to three-fold increased risk of cardiovascular mortality, these results may provide a potential mechanism by which estrogen exerts deleterious effects and potential biomarkers that may indicate a need for additional immunotherapies or alternative estrogen-based therapies in people with HIV.

## Data Availability Statement

The original contributions presented in the study are included in the article/**Supplementary Material**. Further inquiries can be directed to the corresponding author.

## Ethics Statement

The studies involving human participants were reviewed and approved by Institutional Review Board at Ohio State Wexner Medical Center. The patients/participants provided their written informed consent to participate in this study.

## Author Contributions

All authors contributed to experimental design, data analysis, and writing of the manuscript. AK and EB: performed experiments. BH: provided analytical expertise.

## Funding

Research reported in our publication was supported by the National Institutes of Health supported by the National Institute of Allergic and Infectious Disease of the National Institutes of Health under grant numbers P30AI161943, 5R21AI143452 to JEL. This work was also supported, in part, by the National Center for Advancing Translational Sciences of the National Institutes of Health under Grant Numbers TL1TR002735 & UL1TR001450.

## Author Disclaimer

The content is solely the responsibility of the authors and does not necessarily represent the official views of the National Institutes of Health.

## Conflict of Interest

NF has served as a consultant for Gilead.

The remaining authors declare that the research was conducted in the absence of any commercial or financial relationships that could be construed as a potential conflict of interest.

## Publisher’s Note

All claims expressed in this article are solely those of the authors and do not necessarily represent those of their affiliated organizations, or those of the publisher, the editors and the reviewers. Any product that may be evaluated in this article, or claim that may be made by its manufacturer, is not guaranteed or endorsed by the publisher.

## Abbreviations

TW, Transgender women; CM, Cisgender men; CW, Cisgender women; FHT, Feminizing hormone therapy; 17B, 17-β estradiol; 17A, 17-α ethinylestradiol; HIV-, HIV negative; HIV+SP, HIV+ Suppressed; HIV+USP, HIV+ Unsuppressed.
