# Supplementary material for: Estrogen May Enhance Toll-Like Receptor 4-Induced Inflammatory Pathways in People With HIV: Implications for Transgender Women on Hormone Therapy
Source: Front Immunol. 2022 Jun 3;13:879600. doi: 10.3389/fimmu.2022.879600 (PMC9205606; doi:10.3389/fimmu.2022.879600)
Supplement: Supplementary file 1 [file Presentation_1.pptx]

## Slide 1
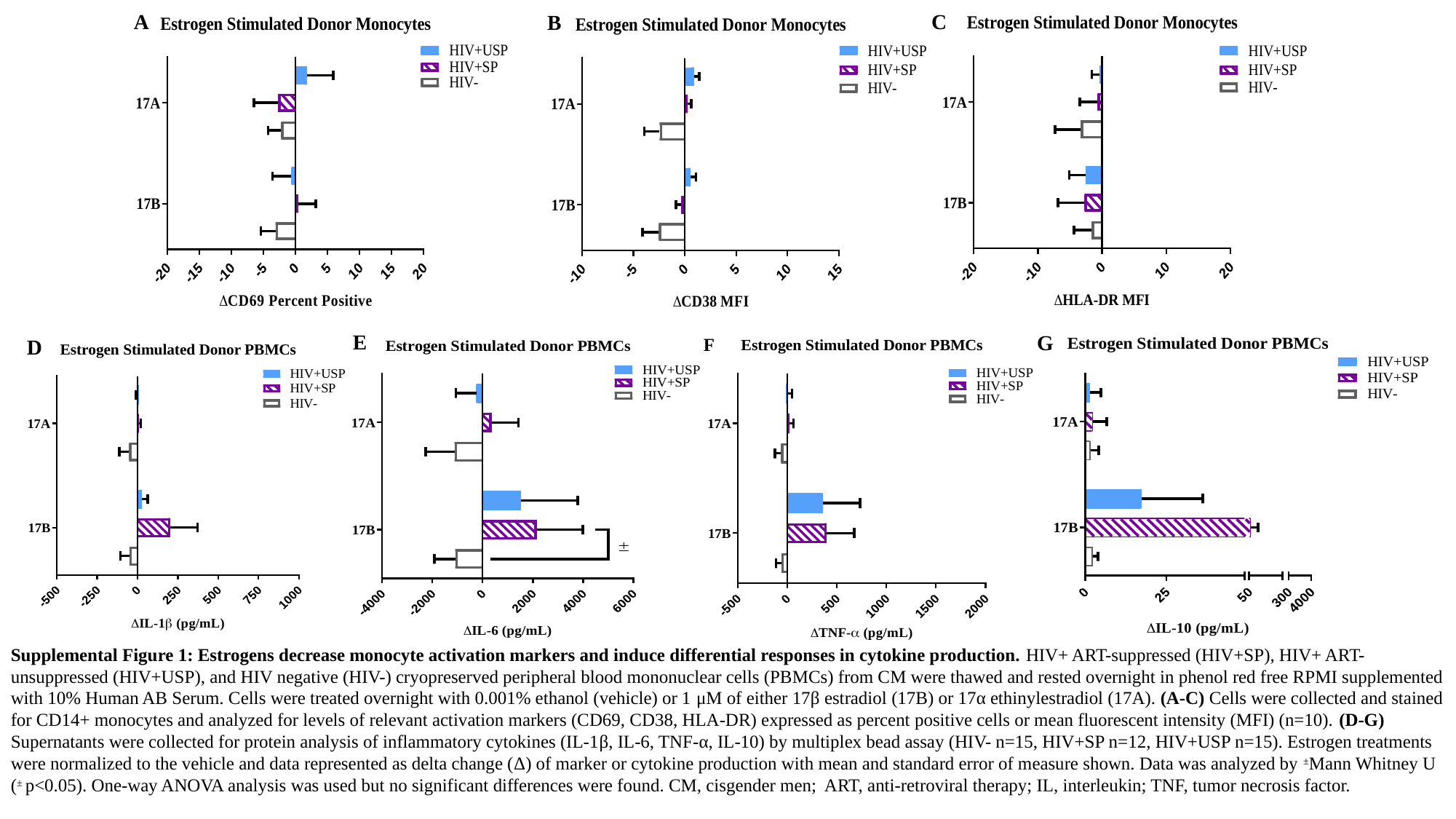

C
A
B
G
E
F
D
Supplemental Figure 1: Estrogens decrease monocyte activation markers and induce differential responses in cytokine production. HIV+ ART-suppressed (HIV+SP), HIV+ ART-unsuppressed (HIV+USP), and HIV negative (HIV-) cryopreserved peripheral blood mononuclear cells (PBMCs) from CM were thawed and rested overnight in phenol red free RPMI supplemented with 10% Human AB Serum. Cells were treated overnight with 0.001% ethanol (vehicle) or 1 μM of either 17β estradiol (17B) or 17α ethinylestradiol (17A). (A-C) Cells were collected and stained for CD14+ monocytes and analyzed for levels of relevant activation markers (CD69, CD38, HLA-DR) expressed as percent positive cells or mean fluorescent intensity (MFI) (n=10). (D-G) Supernatants were collected for protein analysis of inflammatory cytokines (IL-1β, IL-6, TNF-α, IL-10) by multiplex bead assay (HIV- n=15, HIV+SP n=12, HIV+USP n=15). Estrogen treatments were normalized to the vehicle and data represented as delta change (Δ) of marker or cytokine production with mean and standard error of measure shown. Data was analyzed by ±Mann Whitney U (± p<0.05). One-way ANOVA analysis was used but no significant differences were found. CM, cisgender men; ART, anti-retroviral therapy; IL, interleukin; TNF, tumor necrosis factor.

## Slide 2
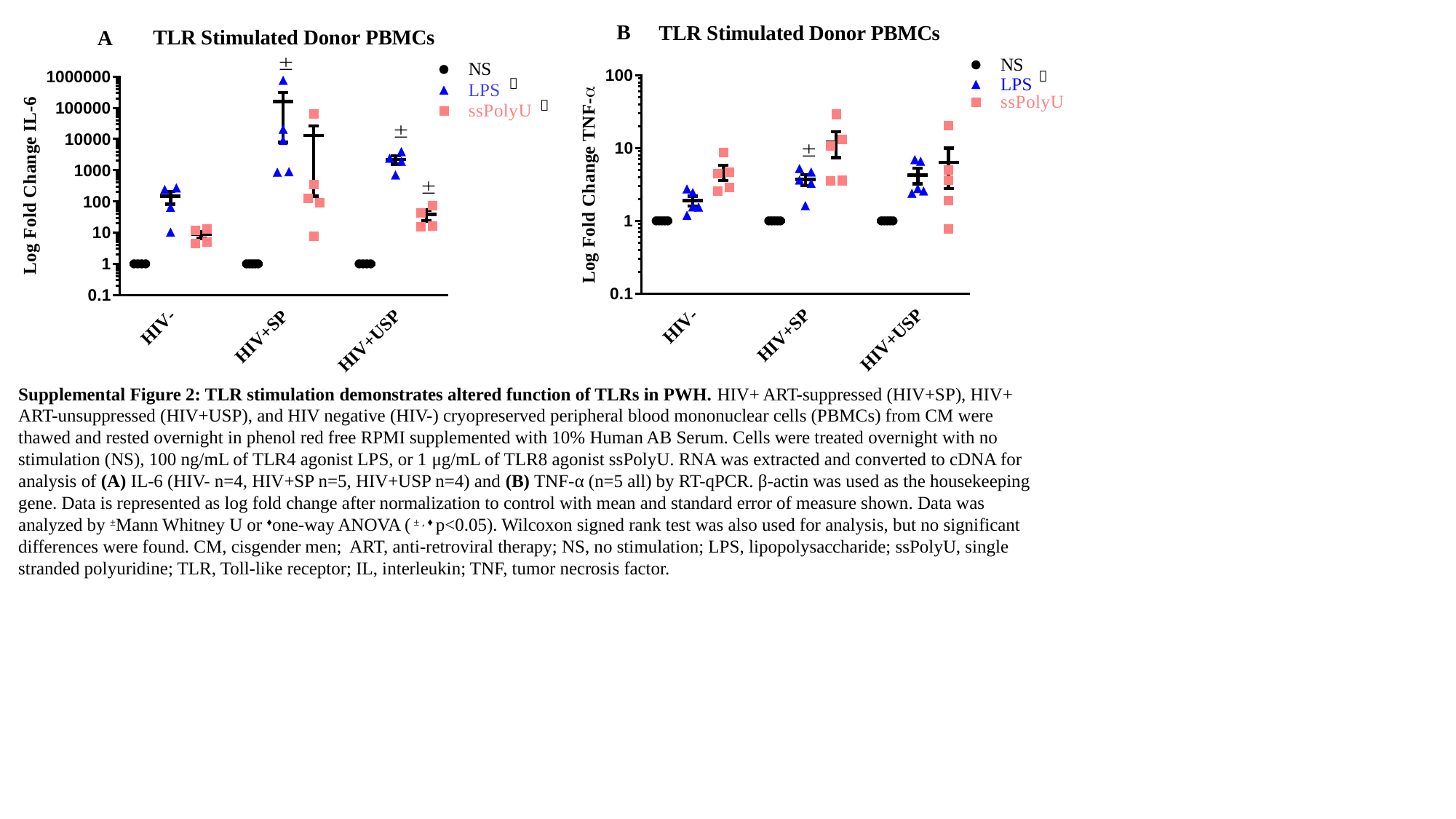

B
A
Supplemental Figure 2: TLR stimulation demonstrates altered function of TLRs in PWH. HIV+ ART-suppressed (HIV+SP), HIV+ ART-unsuppressed (HIV+USP), and HIV negative (HIV-) cryopreserved peripheral blood mononuclear cells (PBMCs) from CM were thawed and rested overnight in phenol red free RPMI supplemented with 10% Human AB Serum. Cells were treated overnight with no stimulation (NS), 100 ng/mL of TLR4 agonist LPS, or 1 μg/mL of TLR8 agonist ssPolyU. RNA was extracted and converted to cDNA for analysis of (A) IL-6 (HIV- n=4, HIV+SP n=5, HIV+USP n=4) and (B) TNF-α (n=5 all) by RT-qPCR. β-actin was used as the housekeeping gene. Data is represented as log fold change after normalization to control with mean and standard error of measure shown. Data was analyzed by ±Mann Whitney U or ♦one-way ANOVA ( ± , ♦ p<0.05). Wilcoxon signed rank test was also used for analysis, but no significant differences were found. CM, cisgender men; ART, anti-retroviral therapy; NS, no stimulation; LPS, lipopolysaccharide; ssPolyU, single stranded polyuridine; TLR, Toll-like receptor; IL, interleukin; TNF, tumor necrosis factor.

## Slide 3
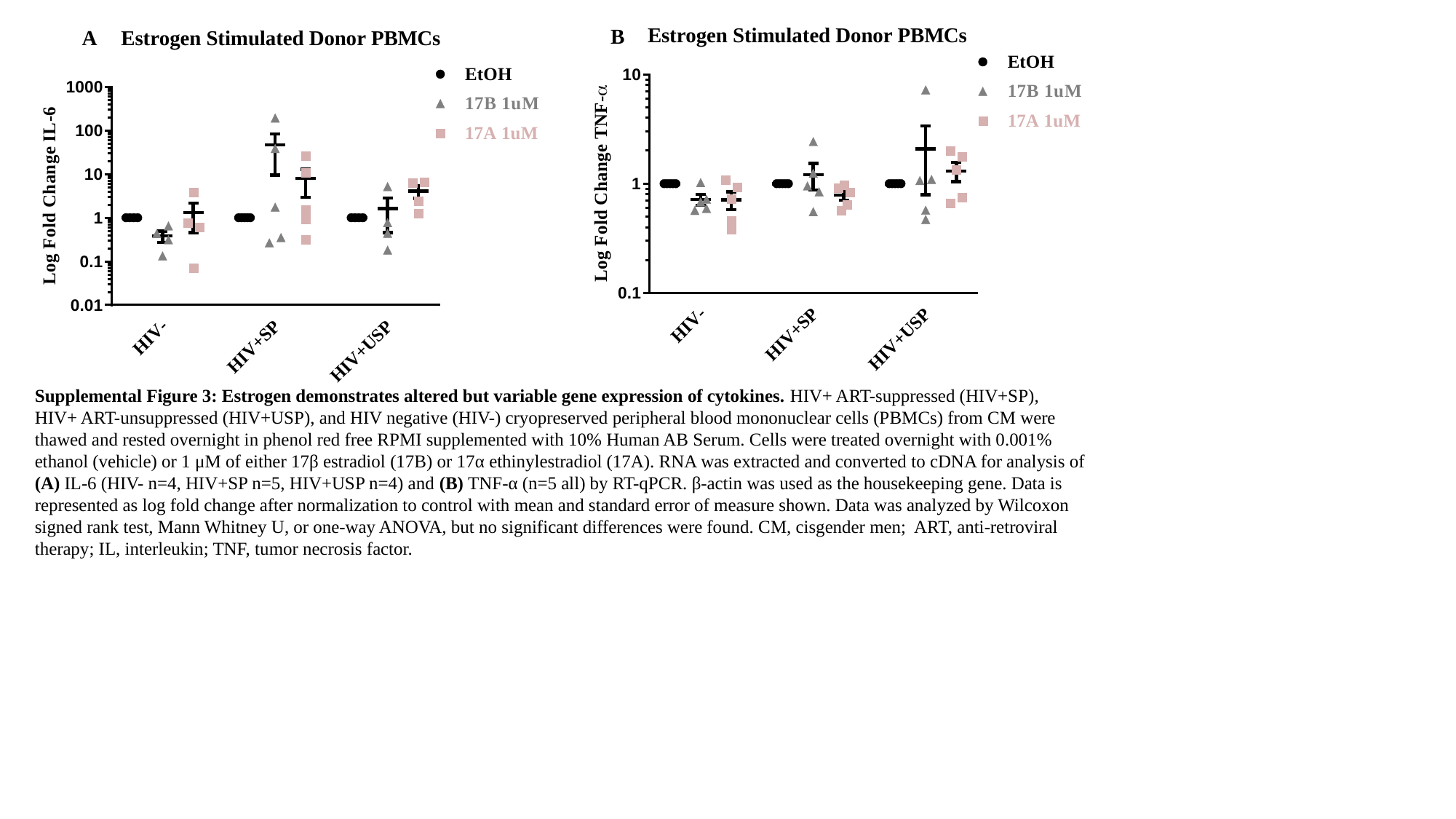

B
A
Supplemental Figure 3: Estrogen demonstrates altered but variable gene expression of cytokines. HIV+ ART-suppressed (HIV+SP), HIV+ ART-unsuppressed (HIV+USP), and HIV negative (HIV-) cryopreserved peripheral blood mononuclear cells (PBMCs) from CM were thawed and rested overnight in phenol red free RPMI supplemented with 10% Human AB Serum. Cells were treated overnight with 0.001% ethanol (vehicle) or 1 μM of either 17β estradiol (17B) or 17α ethinylestradiol (17A). RNA was extracted and converted to cDNA for analysis of (A) IL-6 (HIV- n=4, HIV+SP n=5, HIV+USP n=4) and (B) TNF-α (n=5 all) by RT-qPCR. β-actin was used as the housekeeping gene. Data is represented as log fold change after normalization to control with mean and standard error of measure shown. Data was analyzed by Wilcoxon signed rank test, Mann Whitney U, or one-way ANOVA, but no significant differences were found. CM, cisgender men; ART, anti-retroviral therapy; IL, interleukin; TNF, tumor necrosis factor.

## Slide 4
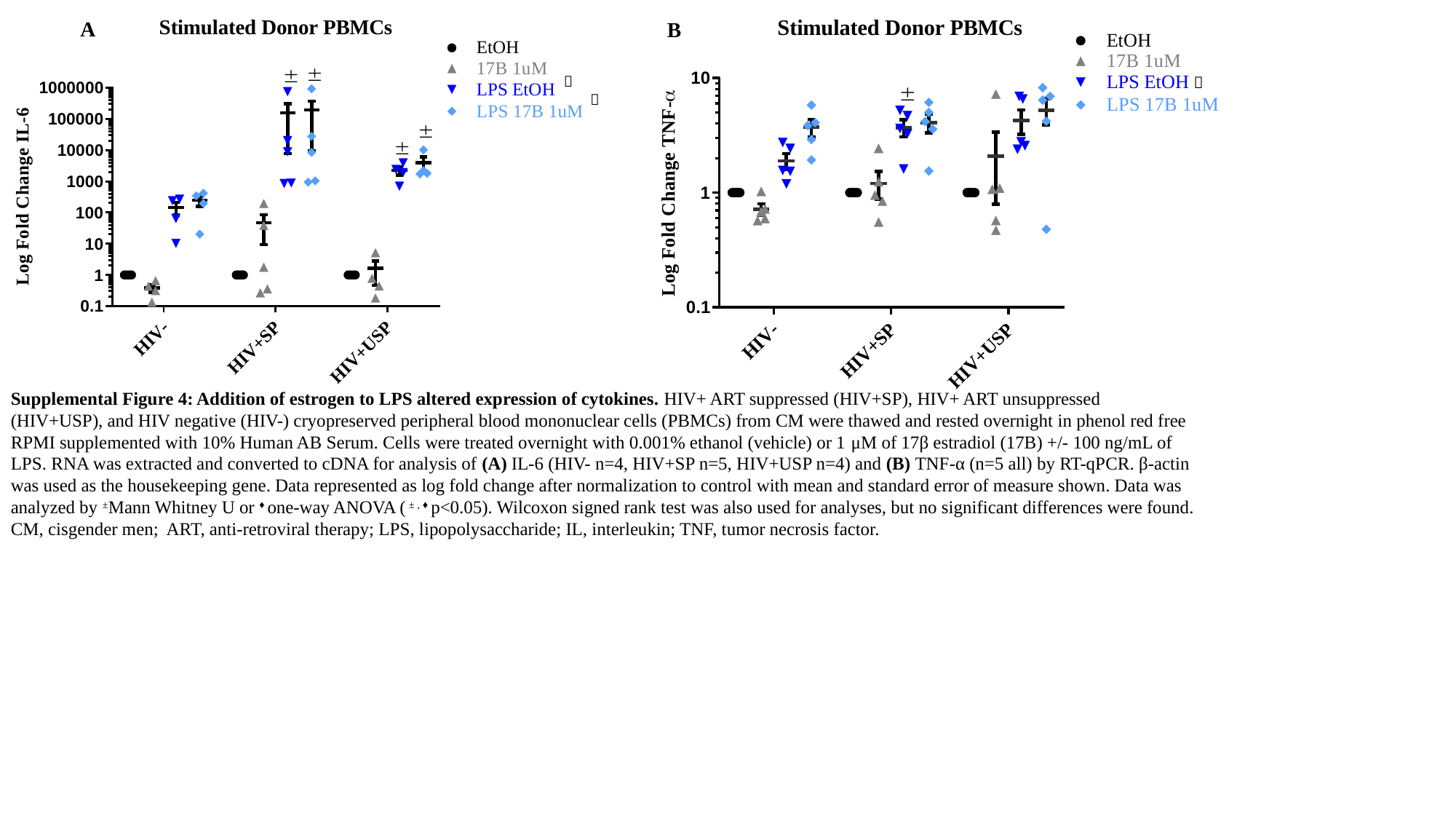

B
A
Supplemental Figure 4: Addition of estrogen to LPS altered expression of cytokines. HIV+ ART suppressed (HIV+SP), HIV+ ART unsuppressed (HIV+USP), and HIV negative (HIV-) cryopreserved peripheral blood mononuclear cells (PBMCs) from CM were thawed and rested overnight in phenol red free RPMI supplemented with 10% Human AB Serum. Cells were treated overnight with 0.001% ethanol (vehicle) or 1 μM of 17β estradiol (17B) +/- 100 ng/mL of LPS. RNA was extracted and converted to cDNA for analysis of (A) IL-6 (HIV- n=4, HIV+SP n=5, HIV+USP n=4) and (B) TNF-α (n=5 all) by RT-qPCR. β-actin was used as the housekeeping gene. Data represented as log fold change after normalization to control with mean and standard error of measure shown. Data was analyzed by ±Mann Whitney U or ♦ one-way ANOVA ( ± , ♦ p<0.05). Wilcoxon signed rank test was also used for analyses, but no significant differences were found. CM, cisgender men; ART, anti-retroviral therapy; LPS, lipopolysaccharide; IL, interleukin; TNF, tumor necrosis factor.

## Slide 5
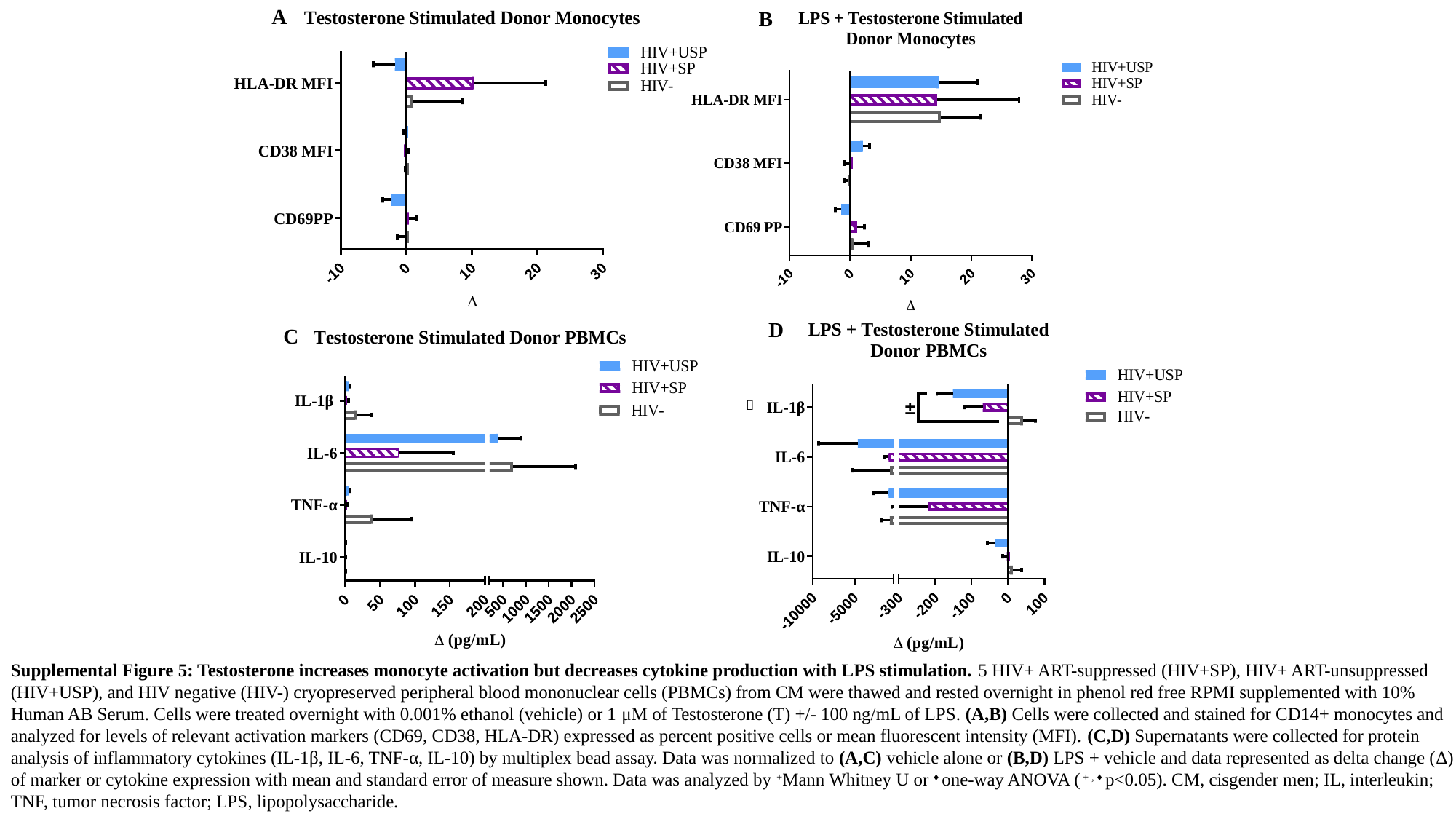

A
B
D
C
Supplemental Figure 5: Testosterone increases monocyte activation but decreases cytokine production with LPS stimulation. 5 HIV+ ART-suppressed (HIV+SP), HIV+ ART-unsuppressed (HIV+USP), and HIV negative (HIV-) cryopreserved peripheral blood mononuclear cells (PBMCs) from CM were thawed and rested overnight in phenol red free RPMI supplemented with 10% Human AB Serum. Cells were treated overnight with 0.001% ethanol (vehicle) or 1 μM of Testosterone (T) +/- 100 ng/mL of LPS. (A,B) Cells were collected and stained for CD14+ monocytes and analyzed for levels of relevant activation markers (CD69, CD38, HLA-DR) expressed as percent positive cells or mean fluorescent intensity (MFI). (C,D) Supernatants were collected for protein analysis of inflammatory cytokines (IL-1β, IL-6, TNF-α, IL-10) by multiplex bead assay. Data was normalized to (A,C) vehicle alone or (B,D) LPS + vehicle and data represented as delta change (Δ) of marker or cytokine expression with mean and standard error of measure shown. Data was analyzed by ±Mann Whitney U or ♦ one-way ANOVA ( ± , ♦ p<0.05). CM, cisgender men; IL, interleukin; TNF, tumor necrosis factor; LPS, lipopolysaccharide.
